# The mechanism of iron binding processes in erionite fibres

**DOI:** 10.1038/s41598-017-01477-x

**Published:** 2017-05-02

**Authors:** Alessandro Pacella, Carlo Cremisini, Elisa Nardi, Maria Rita Montereali, Ida Pettiti, Paolo Ballirano

**Affiliations:** 1 Department of Earth Sciences, Sapienza Università di Roma, Piazzale Aldo Moro 5, I-00185 Roma, Italy; 2 ENEA, C. R. Casaccia, Via Anguillarese 301, I-00123 Roma, Italy; 3 Department of Chemistry, Sapienza Università di Roma, Piazzale Aldo Moro 5, I-00185 Roma, Italy; 4 Rectorial Laboratory Fibres and Inorganic Particulate, Sapienza Università di Roma, Piazzale Aldo Moro 5, I-00185 Roma, Italy

## Abstract

Fibrous erionite-Na from Rome (Oregon, USA) was K-exchanged and characterized from the structural point of view. In addition, the modifications experienced after contact with a Fe(II) source were investigated for evaluating if the large potassium ions, blocking off nearly all the erionite cavity openings, might prevent the Fe(II) binding process, which is currently assumed to be one of the reasons of the toxicity of erionite. The K-exchanged sample had a 95% reduction of the BET surface area indicating that it behaves as a mesoporous material. Exchanged K is segregated at K2 and at OW sites commonly occupied by H_2_O. The latter K cations provide a relevant contribution to the reduction of the surface area. Surprisingly, despite the collapse of its surface area the sample preserves the tendency to bind Fe(II). Therefore, yet in the case of a peculiar and potentially hostile structural environment the Fe(II) ion-exchange process has essentially the same kinetics observed in a typical erionite sample. This is a clear evidence of the very limited effect of the chemical composition of erionite on the Fe(II) binding process and reasonably it does not play a significant role in its toxicity.

## Introduction

Erionite is a fairly common zeolite often occurring with fibrous habit in several geological environments^1^. In the recent years, a relevant number of papers has been published aimed at investigating the chemical and structural properties of this zeolite owing to its well-known link, upon inhalation, with malignant mesothelioma^2^. As a result, erionite has been recently classified as a Group 1 Human-Carcinogen by the International Agency for Research on Cancer (IARC)^3, 4^. Obviously, a crucial point to be addressed is the definition of the mechanism/s inducing carcinogenicity to devise an inactivation route of the fibres. A common view attributes genotoxicity of erionite to the production of reactive oxygen species (ROS) by Fenton reaction^5, 6^. Moreover, it has been shown that erionite is able to induce transformation of human mesothelial cells (MET5-A), but on the other hand, asbestos is unable to cause such transformation^7^. Other factors may be significant in the development of malignant mesothelioma by erionite, such as levels of exposure that are indeed dangerous for the population^8^ as well as genetic predisposition^9^.

Relevantly, erionite is a nominally Fe-free phase but it has been supposed that it is able to fix iron, provided by protein injury, owing to its cation-exchange properties^10^. Recently, it has been shown that erionite can act as a vehiculating media in the human body for iron as several Fe-bearing mineral species (nano-oxydes, sulphates, phyllosilicates) were found to adhere to its surface^11–14^. Therefore, the behaviour of erionite fibres kept in contact with iron sources has been thoroughly investigated to define the fate of both Fe(II) and Fe(III) and in particular the tendency to bind to the surface or to be fixed as extra-framework (EF) cations^15–17^. Results unequivocally showed that Fe(III) is mainly fixed to the surface at relatively high concentration, whereas at low concentrations typical of body fluids it is prevalently segregated at the Ca3 site within the erionite cavity^17^. Differently, Fe(II) is segregated at Ca3 at all concentrations^16^. Unfortunately, this picture indicates that erionite under typical physiological conditions may act as a very efficient ROS generator because iron is located at well-defined crystallographic sites and it possesses a very low nuclearity^18, 19^. Therefore, it appears to be of great interest to investigate different physico-chemical procedures to prevent the access of iron species to the erionite cavity as future roads of (partial) inactivation of such fibres.

Under such aspect, it is worth noting that Eberly^20^ first reported the adsorption properties of erionite and its cation-exchanged forms. He measured a surface area of 203 m^2^ g^−1^ and an effective pore diameter comprised between 4.5 and 5.4 Å, in substantial agreement with the minor and major axes of the largest opening (Ballirano and Cametti^21^: erionite-K 3.50–5.05 Å; Ballirano and Pacella^22^: erionite-Na 3.45–5.05 Å). The K-exchanged form experienced a dramatic reduction of 95% of the surface area resulting in a complete removal of its adsorption properties, apart from a residual adsorptive capacity for H_2_O^20^. Moreover, it has been reported that zeolites having a large K content show reduced catalytic properties^23^.

On this basis, the present work reports the results of a detailed crystal chemical and structural characterization of K-exchanged erionite. In addition, the modifications experienced by the K-exchanged fibres whenever in contact with a ferrous chloride solution were investigated to evaluate if the large potassium ions, blocking off nearly all the adsorption cavities, may prevent the Fe(II) binding process.

## Results

After an enrichment procedure, erionite was K-exchanged by immersion in a concentrated KCl solution (see methods). ICP data indicate that erionite fibres released significant amounts of Na (581 nmol/mg) at the end of the treatment with the KCl solution. SEM-EDS data of the erionite treated with the KCl solution provide clear evidence that the K binding was successful, since the relevant increase of the K content from 2.5 (3) to 5.91 (9) atoms per formula unit (apfu) in the fibres (Table 1). Coherently with ICP data, a marked reduction of the Na content from 3.8 (3) to 1.41 (10) apfu was evidenced. The final average crystal-chemical formula of (Na_1.41_K_5.91_Mg_0.44_)[Al_7.49_Si_28.51_O_72.35_] **·** 30.73H_2_O allows a sample classification as erionite-K.

**Table 1:** Chemical analyses, by SEM-EDS, of both pristine^16^ and KCl treated fibres (3 cycles). Standard deviations are reported in parenthesis.

Oxides (wt.%)	Pristine	KCl treated	KCl treated FeCl_2_
SiO_2_	60.06 (47)	57.35 (14)	58.58 (60)
Al_2_O_3_	12.81 (19)	12.78 (8)	12.82 (17)
Na_2_O	4.03 (27)	1.46 (11)	0.67 (27)
K_2_O	4.01 (42)	9.32 (13)	6.74 (67)
MgO	0.59 (24)	0.59 (9)	0.75 (22)
CaO	0	0	0
FeO	0	0	1.94 (38)
H_2_O*	18.50	18.50	18.50
Total	100.00	100.00	100.00
Si	28.76 (11)	28.51 (5)	28.62 (10)
Al	7.24 (11)	7.49 (5)	7.38 (10)
Na	3.75 (27)	1.41 (10)	0.63 (29)
K	2.45 (27)	5.91 (9)	4.20 (45)
Mg	0.42 (17)	0.44 (7)	0.55 (15)
Ca	0	0	0
Fe	0	0	0.79 (15)
O	72.10 (14)	72.35 (4)	72.07 (17)
H_2_O	29.60 (17)	30.73 (4)	30.20 (25)
R	0.799 (3)	0.792 (1)	0.795 (3)
M/(M + D)	0.936 (27)	0.944 (9)	0.896 (46)
E%	3.1	−8.6	−1.7
Tot. *s.s*. EF cat. (e^−^)	92.8	133.0	114.0

^*^Estimated.

Fe(II)-loading of the K-exchanged fibres was performed by suspending erionite in a 500 µM FeCl_2_ solution. ICP data reveal that erionite fibres bind 290 (13) nmol/mg of Fe. Notably, the Fe amount acquired by erionite-K is perfectly comparable with that observed by Ballirano *et al*.^16^ for erionite-Na after suspension in the 500 µM FeCl_2_ solution [291 (1) nmol/mg]. ICP analysis of supernatants for EF cation release evidences a significant net release of K [445 (33) nmol/mg], coupled with a very minor release of Na, Ca, and Mg during the suspension of the fibres in the FeCl_2_ solution (Table 2). It should be pointed out that the low Ca release detected is due to the presence of traces of admixed impurities (see methods), being Ca absent in the pristine sample as indicated by SEM-EDS analysis (Table 1). The calculated charge balance evidences that the number of Fe acquired charges is comparable with that of released charges (Supplementary Table S1), highlighting that Fe(II) was mainly acquired by erionite through ion-exchange with the EF cations, as reported by Ballirano *et al*.^16^ (Supplementary Table S1). However, in the case of erionite-K, fibres fix Fe(II) within the structure through an ion-exchange process, mainly involving K, while for erionite-Na the process mainly affected Na (Supplementary Table 2). SEM-EDS analysis of the Fe(II) loaded sample shows a significant reduction of both K and Na content [from 5.91 (9) to 4.2 (4) apfu and from 1.41 (10) to 0.6 (3) apfu, respectively], counterbalanced by an upload of 0.79 (15) apfu Fe(II). However, ICP data indicated only a very marginal Na release (Supplementary Table 2). The observed discrepancy between SEM-EDS and ICP data may be attributed to the relevant Na migration occurring in the external layer of fibres during micro-analysis^17, 24^. Summarizing, the following crystal chemical formula (Mg_0.6_Fe_0.7_Na_1.5_K_4.2_)[Al_7.5_Si_28.5_O_72_]∙ 30.5H_2_O may be retrieved for Fe(II) erionite.

**Table 2:** ICP-OES analyses of the supernatant for cation release (calculated as nmol/mg sample).

	Pristine (erionite-Na)	KCl treated (erionite-K)
Mg	5 (0)	2 (0)
Ca	14 (1)	1 (0)
Na	473 (2)	5 (0)
K	42 (1)	445 (33)

Data of pristine sample taken from ref. 16. Data report the net cation release obtained by subtracting the cation release obtained at the same pH (ca. 5) in H_2_O.

Pacella *et al*.^17^ indicated for erionite-Na (pristine material) a BET specific surface area, measured in the conventional p/p_0_ 0.05–0.3 range, of 252 (5) m^2^ g^−1^ and of 325 (5) m^2^ g^−1^ in the p/p^0^ 0–0.1 range, typical of microporous materials (Table 3). The BET specific surface area of the K-exchanged samples collapses to about 12 m^2^ g^−1^. It is worth noting that erionite-Na (pristine material) possesses an external surface area of 10.1 (5) m^2^ g^−1^, i.e. a value very close to the BET surface area of K-exchanged samples (erionite-K), indicating that upon treatment the sample behaves exclusively as a mesoporous material. The total volume of pores decreases from 0.151 cm^3^ g^−1^ of the pristine sample (of which 0.130 cm^3^ g^−1^ attributable to micropores) to ca. 0.02 cm^3^ g^−1^. The Fe(II) loaded sample exhibited an increase of the BET specific surface area to 73.7 (17) m^2^ g^−1^ significantly smaller than that of the pristine sample. In addition, the total volume of pores slightly increases with respect to the K-exchanged samples to 0.0495 cm^3^ g^−1^.

**Table 3:** Results of N_2_ adsorption analyses.

Sample	BET surface p/p^0^ 0.05–0.3 (m^2^ g^−1^)	BET surface p/p^0^ 0.0–0.1 (m^2^ g^−1^)	Pore volume overall (cm^3^ g^−1^)
Pristine*	252 (5)	325 (5)	0.151 (*0.130*)
KCl1	12.0 (5)		0.0213
KCl3	11.0 (5)		0.0195
Fe-exchanged	73.7 (17)		0.0495
Fe-exchanged*	308 (7)	393 (4)	0.182 (*0.151*)

In italics, in parenthesis, volume of micropores. Data of the pristine sample were taken from ref. 23. KCl1 and KCl3 refer to fibres subjected to, respectively, 1 and 3 KCl treatments (see Materials and Methods for details).

Note: *Sample pre-treated at 65 °C for 15 hour and then at 200 °C for 2 hours.

**Figure 1:**
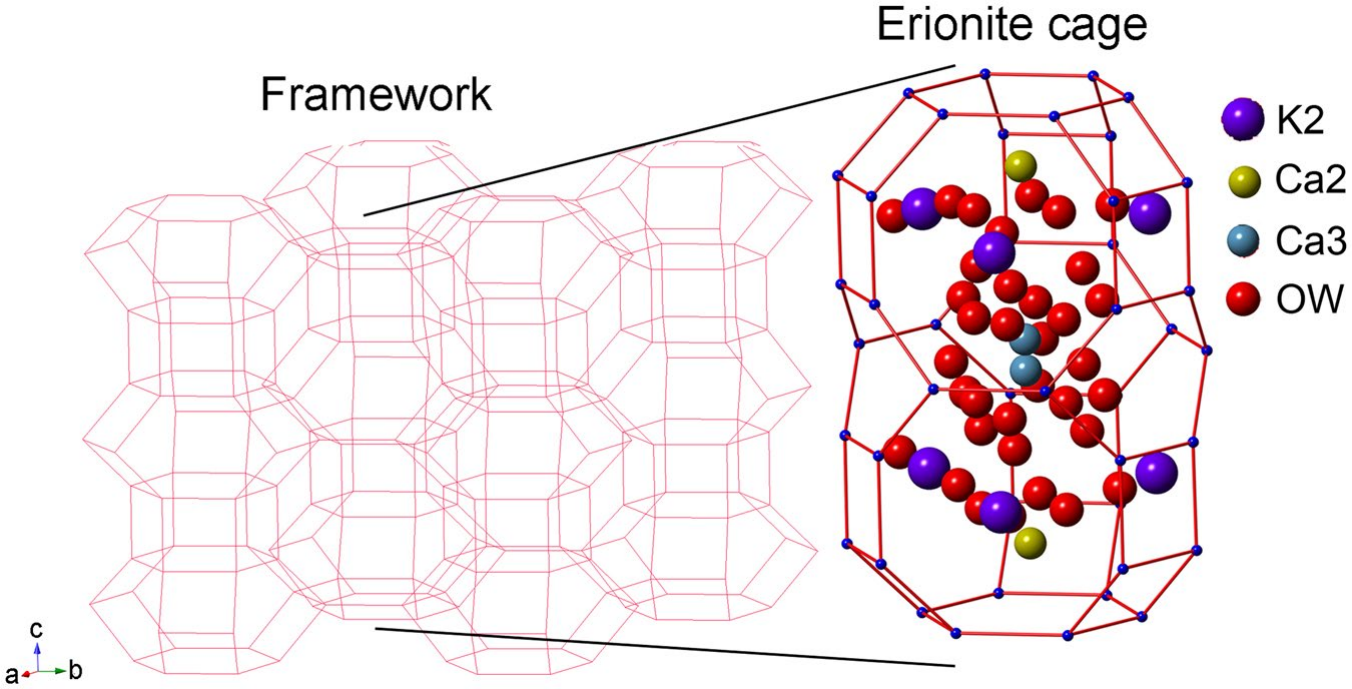
Drawing of (left) the framework of the K-exchanged form of erionite and of (right) the EF cations and water molecules location within the erionite cage. H_2_O residing at OW sites may be partly replaced by K cations.

XRPD data reveal that the cell volume expansion of erionite-K is caused by the *c*-axis expansion only partly counterbalanced by the *a*-axis contraction (Supplementary Table S2). Iron binding produces a further minor contraction of both *a*- and *c*-axis leading to a cell volume smaller than that of erionite-Na (pristine material). The *c/a* ratio passes from ca. 1.138 to ca. 1.140 of the two treated samples (Supplementary Table S2). As expected no significant modifications of the framework (Fig. 1) were detected with the variation of <T-O> bond distances (Supplementary Table S3) and T-O-T bond angles (not shown) being confined within a few standard deviations. The slightly higher R values as calculated from Jones’ determinative curves^25^ with respect to those from SEM-EDS might be related to the effect of protonation at some oxygen sites of the framework progressively occurring during treatments^17^. On the contrary, relevant differences are observed as far as the site scattering (s.s.) of both EF and water molecule sites is referred to (Supplementary Table S4). In the case of the K-exchanged sample we observe a complete depletion of the Ca1 site, which is preferentially occupied by Mg and Na in the pristine sample (erionite-Na) and a very strong reduction of the s.s. at Ca3 (occupied by Na). Conversely, the K2 site (occupied by K) significantly increases its s.s. (Fig. 1). It is worth mentioning that the total EF s.s. drops from ca. 113 e^−^ of the pristine sample to ca. 94 e^−^ of the KCl treated sample apparently indicating a significant cationic discharge during the K binding process. The Fe(II) loading process induces a small refilling of the Ca1 site (ca. 4 e^−^), a significant increase of s.s., from ca. 5 to ca. 20 e^−^ at Ca3, whereas K2 remains substantially unchanged. Concerning the OW sites occupied by H_2_O, the most relevant differences between pristine and KCl treated sample are related to a significant s.s. increase at OW7, OW10 and OW12 and a marked reduction at OW11 in the case of the KCl treated sample (Fig. 1). As a net result, the total OW sites s.s. rises from 245 (11) e^−^ for the pristine to 282 (7) e^−^ for the KCl treated sample. In the case of the Fe(II) loaded sample the s.s. of OW7, OW10 and OW12 reduces back and the total water molecules s.s. decreases to 268 (9) e^−^.

## Discussion

Textural analysis of the KCl treated sample indicates values of the BET surface that are ca. 5% of the pristine material. The corresponding values of ca. 10 m^2^ g^−1^ compare favourably with those observed in both amphibole and chrysotile asbestos (3–20 m^2^ g^−1^) and are consistent with the 7–9 m^2^ g^−1^ reported by Eberly^20^ for erionite-K from Rome, Oregon, USA. At the structural level, the abrupt abatement of the BET surface implies the occlusion of all microporosity. Erionite possesses two apertures that have been considered to act as possible ways for cation mobility, the so-called 8-member (8MR) and 6-member (6MR) rings. The first one, which is the largest, represents the preferential way for any exchange process, whereas the second one, that is shared between the cancrinite and erionite cages, albeit of small dimensions, has been claimed to act as a revolving door for the so-called “internal ionic exchange” mechanism occurring at high temperature^21, 22, 26^. It is worth noting that the K2 site is located approximately at the centre of 8MR. Therefore, the reported increased population at this site implies a more extended occlusion of 8MR leading to a reduction of the BET surface. Despite an exchange process involving the replacement by K (19 electrons) of (mainly) Na (11 electrons) and Mg (12 electrons), the total EF s.s (i.e. the sum of the s.s. of Ca1, Ca2, Ca3, K1, and K2 sites) decreases (Supplementary Table S4). This is a clear indication that a fraction of the EF cations was re-located at sites previously occupied by H_2_O (Fig. 1). This is confirmed by the simultaneous increase of the total OW sites s.s. from 245 (11) e^−^ of the pristine sample to 282 (7) e^−^ for the KCl treated sample mainly justified by a significant s.s. increase at OW7, OW10 and OW12 (Supplementary Table S4). Recent investigations of the thermal behaviour of erionite^21, 22^ have detected relevant s.s. at both OW7 and OW12 at temperatures well exceeding that of dehydration (280–330 °C thermal range). This fact has been attributed to the migration of EF cations toward those sites to obtain a favourable coordination with oxygen atoms of the framework. We may hypothesize the onset of a similar mechanism in the case of the K-binding process. Therefore, the extra 37 e^−^ detected at the water molecule sites of the KCl treated sample with respect to the pristine one may be attributed to EF cations, preferentially K. Summing this extra s.s. to that arising from the EF cations sites a total of 131 e^−^ is obtained in perfect agreement with that determined by SEM-EDS (Table 1). A close inspection of the spatial distribution of the OW sites indicates a few possible coordination types for EF cations residing at (near) the OW7, OW10 and OW12 sites. However, it is expected that the reported fractional coordinates of H_2_O (+cations) sites represent the average position of very close neighbouring sites. Analysis of the s.s. at the various EF cations sites (Table 4) seems to suggest, except for Na, an unusual occurrence of K at Ca2. This hypothesis is supported by the presence of several potential ligands in the 3.1–3.3 Å range (OW12 × 3, OW9 × 3, O5 × 3). Both s.s. and coordination are consistent with the presence of Mg at Ca3 (Table 4). It must be noted that, considering the s.s. from SEM-EDS data and those from Rietveld refinement, there is a remarkably, albeit uncommon, good agreement, the possible mismatch being attributed to alkali-metal migration^23^. In the present case, we may hypothesize that the almost complete pore occlusion caused by the additional K cations prevent alkalies from migration/volatilization.

**Table 4:** Comparison between the site scattering (s.s.) of extra-framework (EF) cation sites calculated from SEM-EDS chemical data (s.s. from partition) and Rietveld refinement (s.s. from Rietveld refinement).

Site	KCl exchanged			Fe-exchanged		
	EF cation	s.s from partition (e^−^)	s.s. from Rietveld refinement (e^−^)	EF cation	s.s from partition (e^−^)	s.s. from Rietveld refinement (e^−^)
Ca1				Mg_0.6_	7.2	4.1 (7)
Ca2	Na_1.5_K_0.6_	27.9	26.1 (8)	Na_0.6(*1.5*)_	6.6 (*16.5*)	24.3 (6)
Ca3	Mg_0.3_	3.6	5.3 (5)	Fe_0.8_	20.8	20.2 (9)
K1	K_2_	38	38	K_2_	38	38
K2	K_1.3_	24.7	24.7 (7)	K_1.1_	20.9	23.1 (6)
OW7–12	K_2_	38	37	K_1.1_	20.9	23
	total	132.2	131 (2)	total	124.3 (*134.2*)	133 (3)

In italics, in parenthesis, the same calculation for an estimated content of Na equal to that of the KCl treated sample, due to the very small release of Na detected by ICP-OES. The low Na content observed by SEM-EDS is due to the renewed Na (and possibly K) cation mobility under the electron beam arising from the leaching of the K cations located at the OW sites.

**Figure 2:**
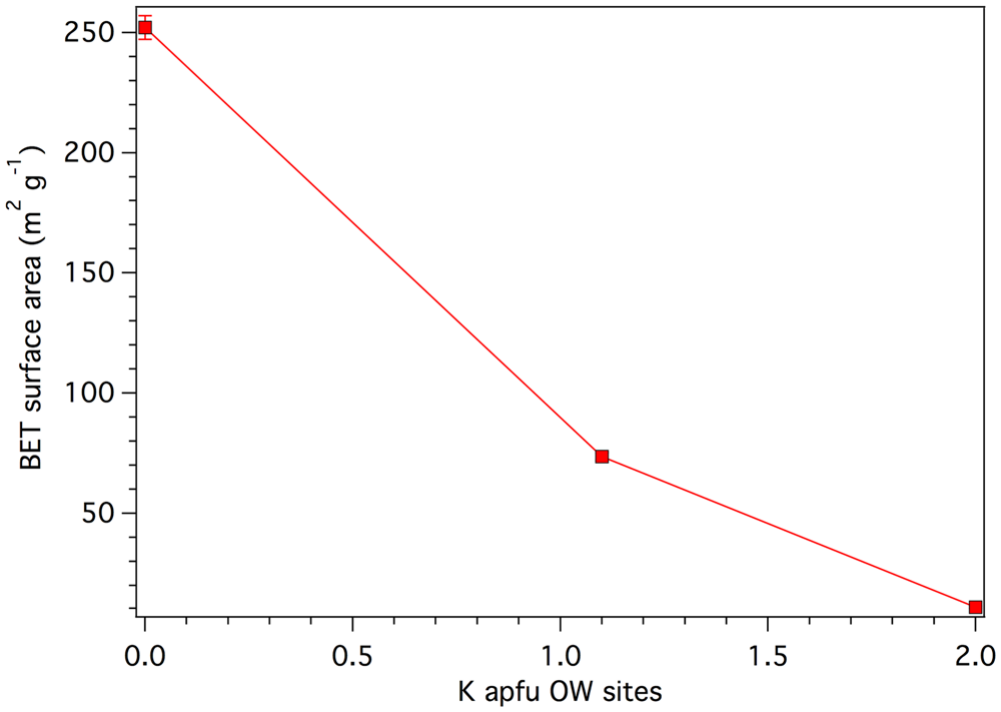
BET surface area as a function of the K content in the OW sites.

It must be pointed out that, despite the collapse of the surface area and the occlusion of all microporosity, the K-exchanged form unfortunately preserved its tendency to bind Fe whenever in contact with a source of Fe(II). Regarding the Fe(II) loaded sample, a significant increase of s.s. at Ca3 may be attributed to the segregation of Fe(II) at this site (Table 4), similarly to the experiments by Ballirano *et al*.^16^. In addition, Ca1 site, previously completely depleted during the K-exchange process, upon iron-loading acquires a s.s. that reasonably matches that of Mg as determined by SEM-EDS. Mobilization of Mg from Ca3 to Ca1 is favoured by a relatively small, non-occurring, distance between the two sites of 1.3 (2) Å. Therefore, we may hypothesize that Mg occupies Ca1 whereas Na is preferentially allocated at Ca2. However, the calculated s.s. of Ca2 is significantly smaller than that retrieved from the Rietveld refinement (Table 4). We may suggest that the Na content has been underestimated in the SEM-EDS analysis due to the well-known, above mentioned, analytical effects. The absence of a coupled significant reduction of s.s. at K2 indicates that the Fe(II) → K exchange process, devised by ICP analysis, prevalently involves K ions located at the OW sites as well as those at Ca2. This hypothesis is confirmed by the reported decrease of the total s.s. of the OW sites from 282 (7) to 268 (9) e^−^ (Supplementary Table S4). Accordingly, the renewed Na (and possibly K) cation mobility under the electron beam can be justified by the leaching of the K cations located at the OW sites. On this basis, hypothesizing the same Na content as in the case of the KCl treated sample (1.5 apfu instead of 0.6), the overall s.s. balance improves significantly. As above mentioned, the BET specific surface area fairly increases with respect to the KCl treated sample passing from ca. 10 to ca. 75 m^2^ g^−1^. Our results showed that this is prevalently due to the release of K ions allocated in the OW sites, that therefore play a primary role in governing the specific surface area of the fibres (Fig. 2).

Notably, the BET specific of the Fe(II) loaded sample measured in the p/p_0_ 0.05–0.3 range [and in the 0–0.1 range], significantly increases from 73.7 (17) m^2^ g^−1^ [and from 75.6 (17) m^2^] to 308 (7) m^2^ g^−1^ if the sample is further pre-treated under vacuum at 200 °C for 1.6 hours (Table 3). The sample heating at this temperature promotes its dehydration and consequently the migration of K hosted from the water molecular OW sites. Accordingly, the sample reacquires the peculiarity of microporous material with the total volume of the sample increasing to 0.182 cm^3^ g^−1^ (of which 0.151 cm^3^ g^−1^ attributable to micropores), value comparable with that measured for the pristine sample (0.151 cm^3^ g^−1^, of which 0.130 cm^3^ g^−1^ attributable to micropores).

## Conclusions

Erionite-Na experienced a dramatic reduction of the BET surface area of ca. 95% upon K-loading indicating that the sample behaves exclusively as a mesoporous material, coherently with literature data. Although the large potassium ions blocked off nearly all the adsorption cavities, erionite-K preserves its capacity of binding Fe(II) in the erionite cavity by ionic exchange, with efficiency comparable to that observed for the pristine sample (erionite-Na). Rietveld refinement highlighted that the K ions residing at the OW sites provide a significant contribution to the reduction of the BET surface area. Coherently, the Fe(II) loaded sample showed a BET specific surface reduced of ca. 25% with respect to that of the pristine sample (erionite-Na). Notably, since the well-known relationship between surface properties of fibres and their interacting ability with biological medium^27–31^, this abrupt reduction supposedly plays a fundamental role in governing the corresponding chemical reactivity of erionite. It is worth noting that our experiments testify the very strong tendency of Fe(II) to bind to the erionite structure owing to its relatively high ionic potential exceeding that of the common EF cations. In fact, it is able to enter even into a very crowded erionite cage hosting additional large K cations at sites previously occupied by water molecules. Therefore, yet in the case of a very peculiar and potentially hostile crystal chemical and structural environment for a Fe(II) ion-exchange process we found essentially the same kinetics observed in a typical erionite sample^16^. This is a clear evidence of the very limited effect of the chemical composition of fibres on the Fe(II) bind process and, accordingly, we may hypothesize that their chemical composition does not play a significant role in inducing toxicity. Present results are of interest in future attempts to engineer alternative roads toward the (partial) inactivation of those fibres.

## Materials and Methods

### Materials

We used erionite-Na from Rome, Oregon, USA that has been previously characterized from the chemical and structural point of view by Ballirano *et al*.^15^. It has a crystal chemical formula (Na_3.75_K_2.45_Mg_0.42_)[Al_7.24_Si_28.76_O_72.10_] **·** 29.60H_2_O. About 5 g of the raw sample were enriched following the protocol devised by Ballirano and Cametti^32^.

### SEM-EDS analysis

Chemical analyses were performed using a Quanta 400 SEM equipped with an EDAX Genesis EDS system (FEI, Hillsboro, OR, USA) and operating at 15 kV accelerating voltage, 11 mm working distance and 0° tilt angle. Chemical data were collected at least at 8 analytical points and the crystal chemical formulae were calculated, after renormalization of the chemical analyses hypothesizing a water content of 18.5 wt.% (corresponding to *ca*. 30 apfu), on the basis of 36 (Si + Al) apfu. The balance error formula E%^33^ and the K content test^34^ were evaluated for selecting the reliable analyses.

### K binding to erionite experiment

K binding was performed following the indications of Eberly^20^ except for rescaling of the quantity of the materials. The KCl solution was obtained by dissolving 75 g of potassium chloride (KCl anhydrous ACS ≥99%, Sigma Aldrich) in 250 mL of deionized water. About 500 mg of erionite were placed in a 50 mL Falcon^TM^ polypropylene tube with 2 mL of KCl solution at 65 °C in an oscillating bath. A non-enriched material was used for a trial to define the best operating conditions for the K-binding procedure. In detail, three different treatments of the fibres with KCl solution were done. In the first one, fibres were stirred with KCl solution for 2 hours. Then, the fibres were separated from the solution by centrifugation at 4000 rpm for 10 min. The solution was filtered with a 0.2 µm membrane filter and stored for ICP-OES analysis. The fibres were recovered on filter and repeatedly rinsed with ultrapure deionized water to remove any residues of Cl ions and K not bound. The washing procedure was repeated until the absence of Cl ions was evidenced by the absence of opalescence in the washing solution after the addition of few drops of a solution of AgNO_3_ (0.1N). At the end of these washing cycles the fibres were dried and stored prior to the BET surface area measurement (KCl1 sample).

In the second experiment, the fibres after having been subjected to the first treatment were added again in a fresh KCl solution and stirred for other two hours. Then, solid and solution were separated following the same procedure described for KCl1 sample and stored for BET (KCl2 sample) and ICP-OES analysis, respectively. In the third experiment, the procedure of KCl treatment was repeated three times (KCl3 sample). A blank procedure, suspending the fibres in deionized water, was also performed. Notably, BET results **(**Table 3 **)** showed no significant differences in the surface area between samples undertaking one (KCl1) or three KCl treatments (KCl3). Accordingly, only one KCl treatment was performed on the enriched material for the K-binding experiment.

### Fe(II) binding to erionite experiment

Fe(II) binding was performed using the enriched erionite sample previously treated with the KCl solution and following the procedure described in Ballirano *et al*.^19^. In detail, 25 mg of erionite fibres were suspended in 25 mL of a 500 µM FeCl_2_ solution, in a 50 mL Falcon^TM^ polypropylene tube. All sample suspensions were prepared in a nitrogen-filled glove-bag to prevent iron oxidation. Later, all the polypropylene tubes, closed and sealed with Parafilm^®^, were removed from the glove-bag and instantly placed in an oscillating bath at 37 °C for 1 hour. After 1 h the tubes were placed again in the oxygen-free glove bag and the supernatant solution was sampled and filtered, using a 0.22 µm nitrocellulose membrane filter, for ICP-OES analysis. The fibres were recovered from the tube on filter, rinsed with ultrapure deionized water to remove any residues of iron not bound, dried and stored under nitrogen prior to the SEM-EDS and XRPD investigations. Blank samples were prepared by suspending fibres in deionized water adjusted to the appropriate pH (ca. 5: i.e. the same pH of the 500 μM FeCl_2_ solution) by adding HCl (Ultrex II, ultrapure reagent, JT Baker). Experiments were done in triplicate.

### ICP-OES analysis

To measure the concentration of released cations from the fibres suspended in both the KCl and FeCl_2_ solutions, the filtered solutions were properly diluted with 1% nitric acid solution and analysed by ICP-OES. All measurements were performed using an Optima 2000 DV ICP-OES spectrometer (Perkin-Elmer, Waltham, MA, USA) equipped with a cross flow nebulizer placed inside a Scott spray chamber. ICP Aristar (BDH) standard solutions in nitric acid for the investigated elements were used to prepare the calibrating solutions for ICP-OES analyses. To ensure adequate quality assurance, the measures of the standard solutions were regularly repeated after the analysis of a defined number of samples. Concerning the Fe(II) binding to erionite experiments, the amount of Fe bound to the fibres (nmol/mg) was calculated by difference between the Fe concentration measured in the initial solution and that measured in the solution after 1 h of sample incubation. The net cation release was calculated by difference between the values measured after suspension in the FeCl_2_ solution and those measured after suspension of fibres in water at the appropriate pH (ca. 5). In order to verify if Fe(II) was acquired by erionite through an ion exchange mechanism, a charge balance was calculated using the same approach of Eborn and Aust^15^. The number of charges of each element was determined multiplying the nanomoles by the valence of the corresponding element and therefore, the net charges released into solution were compared to the total charges acquired in form of Fe.

### Surface area measurements

Surface area, Brunauer–Emmett–Teller (BET) multipoint method^35^, and textural analysis were obtained by N_2_ adsorption/desorption measurements at the liquid nitrogen temperature (−196 °C), using a 3-Flex analyser (Micromeritics, Norcross, GA, USA). Before analysis, samples were pre-treated under vacuum at 65 °C for 15 hours. The pores distribution was determined from the adsorption curve by the Barret–Joyner–Halenda (BJH) method^36^ and from the analysis of the micropore isotherm by the *t*-test^37^ taking the curve of Harkins and Jura^38^. The total pore volume was determined by the rule of Gurvitsch^39^.

### X-ray powder diffraction (XRPD)

XRPD data were collected in transmission mode, using SiO_2_-glass capillaries, on a D8 Advance (Bruker AXS, Karlsruhe, Germany) equipped with incident-beam focussing Göbel mirrors and a PSD VÅntec-1. XRPD patterns were collected in the 6–145° 2θ angular range, 0.022° 2θ step-size and 10 s counting time. Data were evaluated by a mixed Rietveld/Pawley method described in ref. 26 using Topas V.4.2^40^. Starting structural data were taken from Ballirano *et al*.^16^ and refinement procedures follow Ballirano^41^. A content of chabazite (Ca_2_Al_4_Si_8_O_24_ ∙ 12H_2_O) and quartz (SiO_2_) of ca. 3 and 0.1 wt.% have been calculated by the Rietveld method, respectively, and ≪5 wt.% of nontronite has been estimated. Miscellaneous data of the refinements are reported as Supplementary Table S5. Conventional Rietveld plots are reported as Supplementary Figure S1.

## Electronic supplementary material

Supplementary material
