# Supplementary material for: The mechanism of iron binding processes in erionite fibres
Source: Sci Rep. 2017 May 2;7:1319. doi: 10.1038/s41598-017-01477-x (PMC5431018; doi:10.1038/s41598-017-01477-x)
Supplement: Supplementary file 1 — Supplementary material [file 41598_2017_1477_MOESM1_ESM.docx]

**The mechanism of iron binding processes in erionite fibres**

Alessandro Pacella^a^, Carlo Cremisini^b^, Elisa Nardi^b^, Maria Rita Montereali^b^, Ida Pettiti^c^, Paolo Ballirano^a,d^

^a^Department of Earth Sciences, Sapienza Università di Roma, Piazzale Aldo Moro 5, I-00185 Roma, Italy

^b^ENEA, C.R. Casaccia via Anguillarese 301, I-00123 S. Maria di Galeria, Roma, Italy

^c^ Department of Chemistry, Sapienza Università di Roma, Piazzale Aldo Moro 5, I-00185 Roma, Italy

^d^Rectorial Laboratory Fibres and Inorganic Particulate, Sapienza Università di Roma, Piazzale Aldo Moro 5, I-00185 Roma, Italy

**Supplementary material**

This file includes the electronic supporting information of the paper entitled “*The mechanism of iron binding processes in erionite fibres*”.

***List of Figures:***

**Figure S1**. Magnified view (6-80°2θ) of the Rietveld plots of **a**) K-exchanged erionite and **b**) FeCl_2_ treated erionite samples. Insets: high-angle portion of the Rietveld Plots. Blue dots refer to the experimental pattern, red continuous to the calculated pattern and grey continuous to the difference plot. Markers report the position of calculated Bragg reflections of, from above to below, erionite, chabazite, quartz and nontronite [two main peaks at 19.65 (010) and 34.37°2θ (110)].

***List of Tables***

**Table S1.** Charge balance involved in the process of Fe(II) loading. For each element, the net charge released was calculated by multiplying the net cation release by the valence of the cation. In addition, the acquired charge was calculated by multiplying values of bound Fe(II) by 2. The contribution of Ca, arising from impurities, has not been taken into account. Data of pristine sample taken from reference 16.

**Table S2.** Comparison of cell parameters and volume of pristine and KCl treated fibres. Data of pristine sample taken from reference 16.

**Table S3.** Si, Al partition at T1 and T2 sites and calculation of the R ratio following Jones’ determinative curves^24^. Data of pristine sample taken from reference 16.

**Table S4.** Fractional coordinates, isotropic displacement parameters (B_iso_) site occupancy fractions (sof), site scattering from refinement (s.s. ref.), of EF cations and water molecules, estimated s.s. of EF cations residing at OW sites, and estimated total EF s.s. of KCl exchanged and FeCl_2_ treated erionite samples.

The s.s. ref. were obtained by multiplying site multiplicity, sof and scattering power of each site. Scattering power of Na was used for Ca1, Ca2 and Ca3; K for K1 and K2; O for OW sites.

The estimated s.s. of EF in OW7-OW12 was obtained by comparing the s.s. of the OW sites of the pristine with those of the treated samples (see text for explanation).

The estimated total EF s.s. was obtained by summing the estimated s.s. of EF in OW7-OW12 to the Σ_EF cations s.s._

**Table S5.** Experimental details and miscellaneous data of the mixed Rietveld/Pawley refinement. A nontronite content << 5 wt.% has been estimated. Statistical parameters as defined in Young [1].

**Figure S1**. Magnified view (6-80°2θ) of the Rietveld plots of **a**) K-exchanged erionite and **b**) FeCl_2_ treated erionite samples. Insets: high-angle portion of the Rietveld Plots. Blue dots refer to the experimental pattern, red continuous to the calculated pattern and grey continuous to the difference plot. Markers report the position of calculated Bragg reflections of, from above to below, erionite, chabazite, quartz and nontronite [two main peaks at 19.65 (010) and 34.37°2θ (110)].


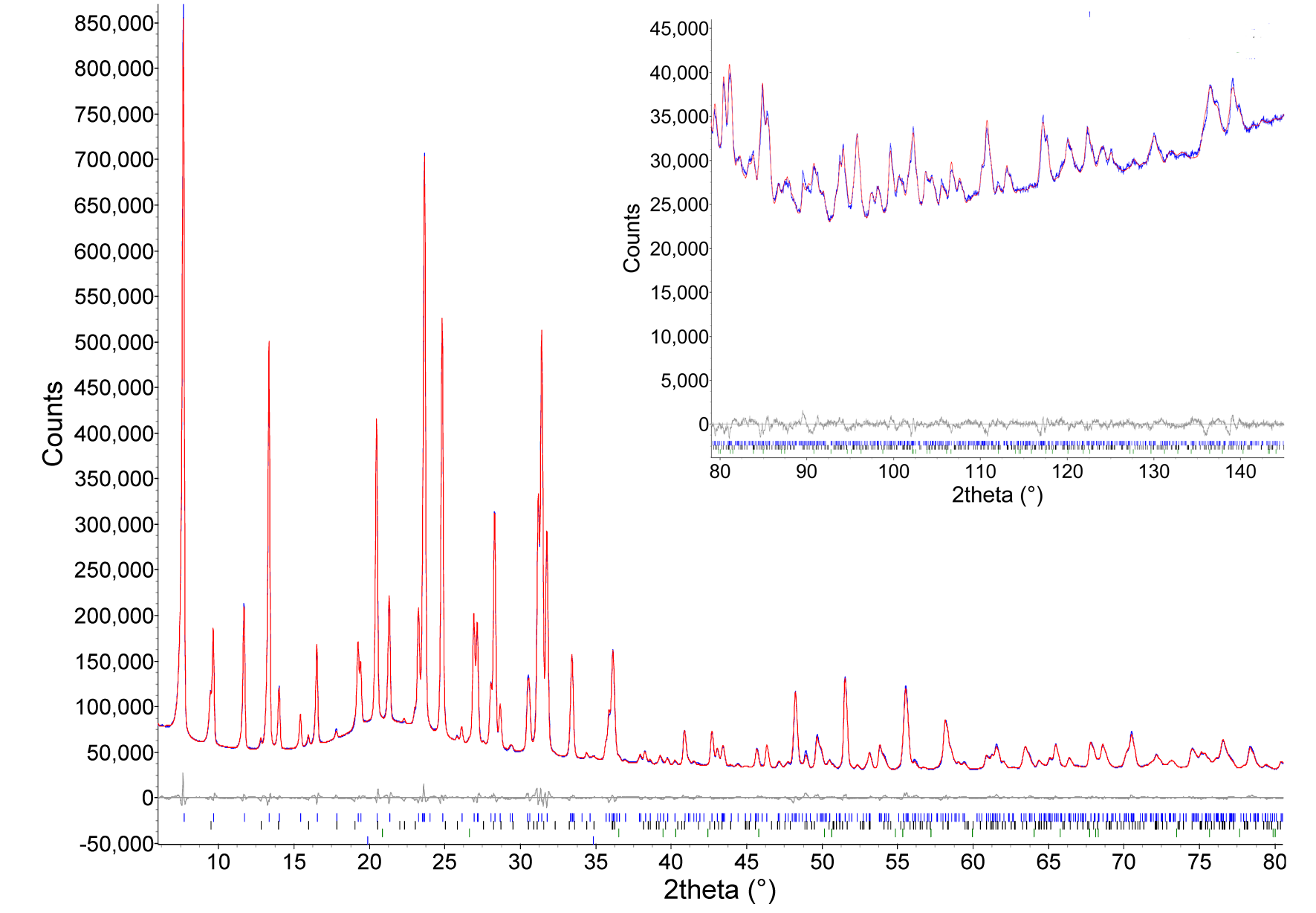


a)


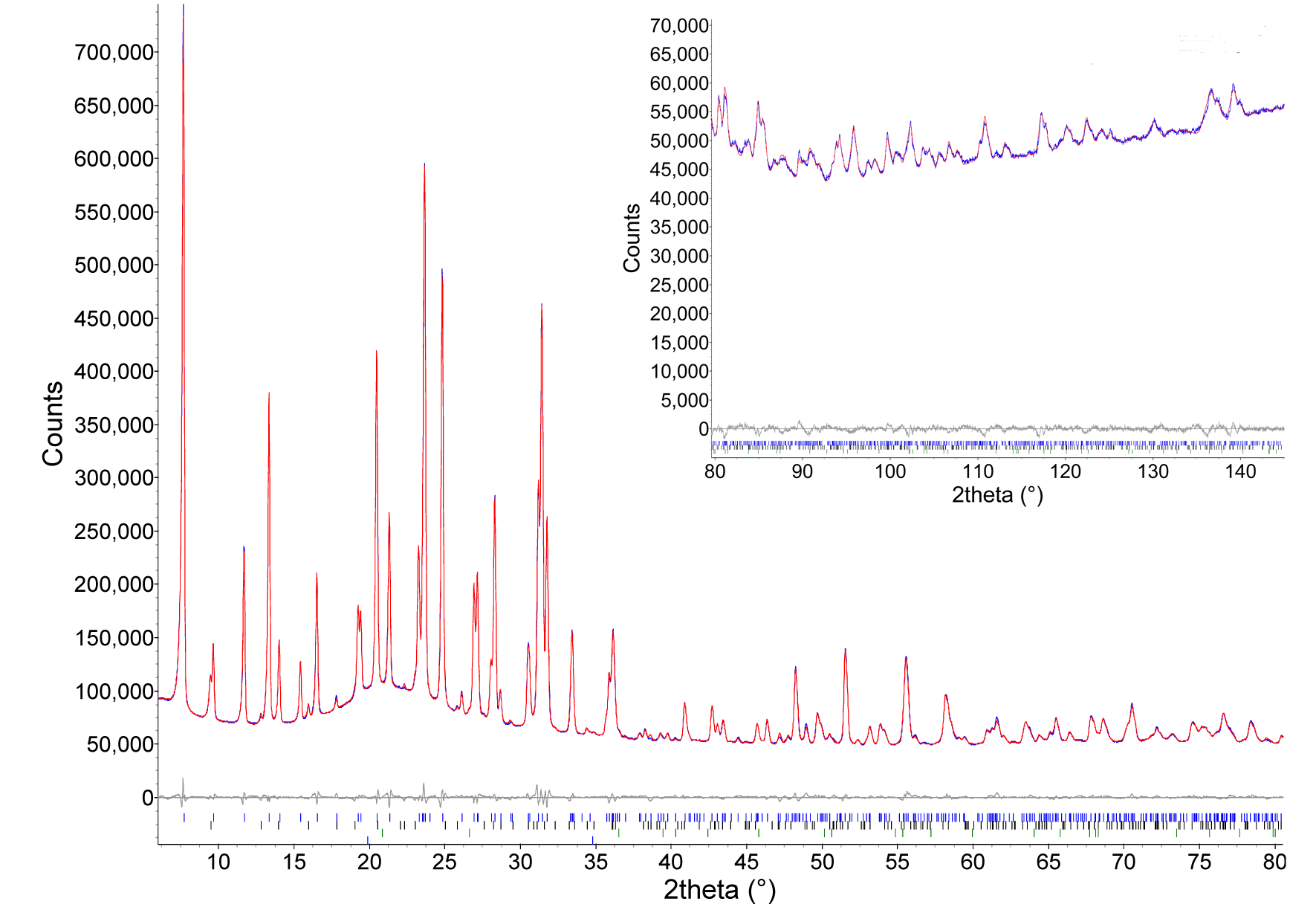


b)

**Table S1.** Charge balance involved in the process of Fe(II) loading. For each element, the net charge released was calculated by multiplying the net cation release by the valence of the cation. In addition, the acquired charge was calculated by multiplying values of bound Fe(II) by 2. The contribution of Ca, arising from impurities, has not been considered. Data of pristine sample taken from reference 16.

| Net charge (e^-^) | Pristine  (erionite-Na) | KCl treated  (erionite-K) |
| --- | --- | --- |
| Total released EF charges | 525(3) | 454(33) |
| Acquired charges (Fe) | 582(3) | 579(26) |
| % Fe(II) as EF cation | 90 | 78 |

**Table S2.** Comparison of cell parameters and volume of pristine and KCl treated fibres. Data of pristine sample taken from reference 16.

| Sample | *a* (Å) | *c* (Å) | *c/a* | Volume (Å^3^) |
| --- | --- | --- | --- | --- |
| Pristine | 13.23057(9) | 15.06026(11) | 1.1383 | 2283.07(4) |
| KCl exchanged | 13.22555(8) | 15.07846(10) | 1.1401 | 2284.10(3) |
| Fe-exchanged | 13.22153(9) | 15.07314(10) | 1.1400 | 2281.91(3) |

**Table S3.** Si, Al partition at T1 and T2 sites and calculation of the R ratio following Jones’ determinative curves^24^. Data of pristine sample taken from reference 16.

|  | <T1-O> | <T2-O> | T1 | T2 | T | R |
| --- | --- | --- | --- | --- | --- | --- |
| Pristine | 1.6276 | 1.6444 | Si_20.31_Al_3.69_ | Si_8.86_Al_3.14_ | Si_29.17_Al_6.83_ | 0.810 |
| KCl exchanged | 1.6274 | 1.6461 | Si_20.35_Al_3.65_ | Si_8.73_Al_3.27_ | Si_29.08_Al_6.92_ | 0.808 |
| Fe-exchanged | 1.6252 | 1.6504 | Si_20.68_Al_3.32_ | Si_8.40_Al_3.60_ | Si_29.08_Al_6.92_ | 0.808 |

**Table S4**. Fractional coordinates, isotropic displacement parameters (B_iso_) site occupancy fractions (sof), site scattering from refinement (s.s. ref.) of EF cations and water molecules, estimated s.s. of EF cations residing at OW sites, and estimated total EF s.s. of KCl exchanged and FeCl_2_ treated erionite samples.

The s.s. ref. were obtained by multiplying site multiplicity, sof and scattering power of each site. Scattering power of Na was used for Ca1, Ca2 and Ca3; K for K1 and K2; O for OW sites.

The estimated s.s. of EF in OW7-OW12 was obtained by comparing the s.s. of the OW sites of the pristine with those of the treated samples (see text for explanation).

The estimated total EF s.s. was obtained by summing the estimated s.s. of EF in OW7-OW12 to the Σ_EF cations s.s._

|  | **KCl exchanged** | | | | | |  |
| --- | --- | --- | --- | --- | --- | --- | --- |
| Site | *x* | *y* | *z* | B_iso_ (Å^2^) | sof | s.s. ref. (e^-^) | |
| Framework |  |  |  |  |  |  | |
| Si1 (Si_0.852_Al_0.148_) | 0.23451(9) | 0.00027(13) | 0.10426(5) | 0.722(16) | 1 |  | |
| Si2 (Si_0.728_Al_0.272_) | 0.09325(13) | 0.42490(14) | ¼ | 0.722(16) | 1 |  | |
| O1 | 0.34799(17) | 0.02469(17) | 0.65990(16) | 1.07(4) | 1 |  | |
| O2 | 0.09799(13) | 2*x* | 0.1243(2) | 1.07(4) | 1 |  | |
| O3 | 0.12601(12) | 2*x* | 0.6345(2) | 1.71(6) | 1 |  | |
| O4 | 0.26888(18) | 0 | 0 | 1.07(4) | 1 |  | |
| O5 | 0.2312(2) | 2*x* | ¼ | 1.71(6) | 1 |  | |
| O6 | 0.4592(2) | 2*x* | ¼ | 1.71(6) | 1 |  | |
| EF Cations |  |  |  |  |  |  | |
| Ca2 | 1/3 | 2/3 | 0.1021(12) | 19.8(10) | 0.594(18) | 26.1(8) | |
| Ca3 | 1/3 | 2/3 | 0.700(5) | 19.8(10) | 0.066(7) | 5.3(5) | |
| K1 | 0 | 0 | ¼ | 2.48(7) | 1 | 38 | |
| K2 | 1/2 | 0 | 0 | 2.48(7) | 0.217(6) | 24.7(7) | |
| Σ_EF cations s.s._ |  |  |  |  |  | 94(2) | |
| OW7 | 0.2387(4) | 2*x* | ¾ | 8.06(13) | 0.942(15) | 45.2(7) | |
| OW8 | 0.2648(8) | 2*x* | 0.0299(11) | 16.1(2) | 0.395(7) | 37.9(7) | |
| OW9 | 0.4073(7) | 2*x* | 0.922(2) | 16.1(2) | 0.518(16) | 49.7(15) | |
| OW10 | 0.4217(7) | 2*x* | 0.6473(14) | 16.1(2) | 0.57(2) | 54.3(19) | |
| OW11 | 0.2606(17) | 2*x* | 0.668(3) | 16.1(2) | 0.243(10) | 23.3(9) | |
| OW12 | 0.4594(5) | 2*x* | 0.0186(7) | 16.1(2) | 0.747(9) | 71.7(9) | |
| Σ_water mol. s.s_ |  |  |  |  |  | 282(7) | |
| Estimated s.s. of EF in OW7-12 |  |  |  |  |  | 37 | |
| Estimated total EF s.s. |  |  |  |  |  | 131 | |

|  | **KCl exchanged FeCl_2_** | | | | | |  |
| --- | --- | --- | --- | --- | --- | --- | --- |
| Site | *x* | *y* | *z* | B_iso_ | sof | s.s. ref. | |
| Framework |  |  |  |  |  |  | |
| Si1 (Si_0.862_Al_0.138_) | 0.23457(10) | 0.00038(14) | 0.10434(6) | 0.711(18) | 1 |  | |
| Si2 (Si_0.700_Al_0.300_) | 0.09332(15) | 0.42456(15) | ¼ | 0.711(18) | 1 |  | |
| O1 | 0.34799(17) | 0.02470(19) | 0.65923(17) | 1.02(5) | 1 |  | |
| O2 | 0.09816(13) | 2*x* | 0.1249(2) | 1.02(5) | 1 |  | |
| O3 | 0.12580(13) | 2*x* | 0.6338(2) | 1.90(7) | 1 |  | |
| O4 | 0.26842(18) | 0 | 0 | 1.02(5) | 1 |  | |
| O5 | 0.2320(2) | 2*x* | ¼ | 1.90(7) | 1 |  | |
| O6 | 0.4592(2) | 2*x* | ¼ | 1.90(7) | 1 |  | |
| EF Cations |  |  |  |  |  |  | |
| Ca1 | 1/3 | 2/3 | 0.888(11) | 15.7(8) | 0.051(9) | 4.1(7) | |
| Ca2 | 1/3 | 2/3 | 0.1049(11) | 15.7(8) | 0.553(14) | 24.3(6) | |
| Ca3 | 1/3 | 2/3 | 0.7013(18) | 15.7(8) | 0.252(11) | 20.2(9) | |
| K1 | 0 | 0 | ¼ | 2.53(9) | 1 | 38 | |
| K2 | 1/2 | 0 | 0 | 2.53(9) | 0.202(6) | 23.1(6) | |
| Σ_EF cations s.s._ |  |  |  |  |  | 110(3) | |
| OW7 | 0.2405(8) | 2*x* | ¾ | 8.29(14) | 0.63(4) | 23.1(6) | |
| OW8 | 0.2651(7) | 2*x* | 0.0233(13) | 16.6(3) | 0.423(7) | 40.6(7) | |
| OW9 | 0.4085(10) | 2*x* | 0.925(2) | 16.6(3) | 0.537(18) | 51.6(17) | |
| OW10 | 0.4242(8) | 2*x* | 0.6452(17) | 16.6(3) | 0.51(2) | 49(2) | |
| OW11 | 0.2505(16) | 2*x* | 0.688(3) | 16.6(3) | 0.35(2) | 33.7(19) | |
| OW12 | 0.4550(6) | 2*x* | 0.0218(9) | 16.6(3) | 0.656(10) | 63.0(10) | |
| Σ_water mol. s.s._ |  |  |  |  |  | 282(7) | |
| Estimated EF in OW7-12 |  |  |  |  |  | 23 | |
| Estimated total EF s.s. |  |  |  |  |  | 133 | |

**Table S5**. Experimental details and miscellaneous data of the mixed Rietveld/Pawley refinement. A nontronite content << 5 wt.% has been estimated. Statistical parameters as defined in Young [1].

| **Samples** | **KCl exchanged** | **KCl exchanged FeCl_2_** |
| --- | --- | --- |
| Instrument | Bruker AXS D8 Advance | |
| Radiation | CuKα | |
| Primary and secondary radius (mm) | 250 | |
| Detector | PSD VÅNTEC-1 | |
| Sample mount | Rotating capillary (60 r/min) | |
| Incident beam optics | 60 mm focusing multilayer (Göbel) X-ray mirror | |
| Soller slits | Primary beam (2.3°); diffracted beam (radial) | |
| Detector window (°) | 6 | |
| Divergence slits (°) | 0.1 | |
| Sample length = receiving slit length (mm) | 8.9 | |
| Angular range (°2θ) | 6-145 | |
| (sin θ/λ)_max_ (Å^-1^) | 0.619 | |
| Step size (°2θ) | 0.022 | |
| Counting time (s) | 10 | |
| Chabazite (wt.%) | 2.75(8) | 3.08(8) |
| Quartz (wt.%) | 0.10(1) | 0.11(1) |
| R_Bragg_ (%) | 0.65 | 0.41 |
| R_P_ (%) | 1.33 | 0.96 |
| R_WP_ (%) | 1.82 | 1.32 |

**References**

[1] Young, R.A. in: Young R.A. (Ed.) The Rietveld method, Oxford University Press, Oxford, 1993, pp 1-38.
